# Clinical Significance of Histone Deacetylase (HDAC)-1, -2, -4 and -6 Expression in Salivary Gland Tumors

**DOI:** 10.3390/diagnostics11030517

**Published:** 2021-03-14

**Authors:** Despoina Pouloudi, Maria Manou, Panagiotis Sarantis, Nikolaos Tsoukalas, Gerasimos Tsourouflis, Eougken Dana, Michalis V. Karamouzis, Jerzy Klijanienko, Stamatios Theocharis

**Affiliations:** 1 First Department of Pathology, Medical School, National and Kapodistrian University of Athens, 115 27 Athens, Greece; d.v.pouloudi@gmail.com (D.P.); maria_manou@hotmail.com (M.M.); psarantis@med.uoa.gr (P.S.); ntsoukn@yahoo.gr (N.T.); eugenedanas@gmail.com (E.D.); 2 Department of Biological Chemistry, Medical School, National and Kapodistrian University of Athens, 115 27 Athens, Greece; mkaramouz@med.uoa.gr; 3 2nd Department of Propedeutic Surgery, School of Medicine, National and Kapodistrian, University of Athens, 115 27 Athens, Greece; gtsourouflis@med.uoa.gr; 4 Department of Pathology, Institut Curie, 75248 Paris, France; jerzy.klijanienko@curie.fr

**Keywords:** HDAC, salivary gland cancer, immunohistochemistry, prognosis

## Abstract

Salivary gland tumors (SGTs) comprise a group of rare neoplasms. Locally aggressive, recurrent and/or metastatic SGTs are notorious for their resistance to systemic therapy, making the need for carefully designed, prospective and randomized trials with useful predictive markers mandatory to define new effective therapeutic protocols. Histone Deacetylases (HDACs), are thought to play a crucial role in carcinogenesis. They affect the DNA structure, being also able to regulate its transcription, repair, and replication. This study aimed to evaluate—to our knowledge for the first time—the HDAC-1, -2, -4 and -6 immunohistochemical expression in SGTs and their potential use as prognostic biomarkers. Medical records and archival histopathological material of 58 (36 benign and 22 malignant) SGT patients were included in this study. The *H*-score was statistically correlated with the clinicopathological characteristics for all cases and patients’ survival rate in malignant SGTs. HDAC-2 positivity was significantly associated with more prolonged overall survival (OS) of patients with malignant SGTs (*p* = 0.028), while HDAC-2 positivity and no HDAC-6 expression were associated with prolonged OS of patients with HG malignant SGT (*p* = 0.003 and *p* = 0.043, respectively). Additionally, a high HDAC-2 H-score was significantly associated with longer OS for HG malignant SGT patients (*p* = 0.027). In our study, HDAC-2 expression is a marker for good prognosis, whereas HDAC-6 expression indicated poor prognosis; thus, an inhibitor of HDAC-6 may be used to improve patients’ survival.

## 1. Introduction

Salivary gland tumors (SGTs) comprise a group of rare neoplasms, accounting for 3% to 10% of all head and neck tumors, characterized by histological diversity with—occasionally extensive—overlapping morphology and a broad spectrum of clinical behavior. The number of carcinoma types has increased from 5 in the 1972 WHO classification edition, to 22 in the 2017 one, and, in the same period, the number of benign tumors has increased from 4 to 11 [1,2]. Therefore, diagnostic and classification challenges are not uncommon [3]. Current research aims towards new immunohistochemical markers and molecular techniques to overcome these difficulties [4,5,6,7,8,9]. As far as malignant SGTs are concerned, their annual incidence ranges from 0.5 to 2 per 100,000 internationally [10]. Although 5-year survival is thought to be rather favorable for most of the distinct histologic types of SGT, survival rates seem to drop in the long-term, underlining that malignancy’s lethal effect manifests itself late in the course of the disease [11]. Moreover, locally aggressive, recurrent and/or metastatic SGTs are notorious for their resistance to systemic therapy [12], making the need for carefully designed, prospective and randomized trials with useful predictive markers mandatory to define new effective therapeutic protocols [13,14]. Histone deacetylase inhibitors (HDACIs) are included among the numerous therapeutic regimens for the SGTs currently under study [15].

Nowadays, epigenetic phenomena are considered one of cancer’s hallmarks, including SGTs [16,17]. Among them, post-translational modifications (PTMs) of DNA bounded-histones hold a crucial role in carcinogenesis. Histones are a heterogeneous group of proteins responsible for DNA condensation, representing the basic nucleosome component [18]. They affect the DNA structure being also able to regulate its transcription, repair and replication [18,19]. Histones are composed of a flexible “tail” susceptible to post-translation biochemical modifications, such as acetylation, methylation, ubiquitination and phosphorylation acetylation and methylation represents the most frequently described histones epigenetic modifications [11,18,20]. Histone acetylation on Lys residues, mediated by the histone acetyltransferase enzymes (HATs), is a common type of PTM that is highly reversible by a group of enzymes called Histone Deacetylases (HDACs). Histone deacetylation is associated with closed or repressive chromatin structure. Human HDACs comprise a group of 18 enzymes classified into four classes according to their sequence homology with *S. cerevisiae* yeast proteins: Class I includes HDAC-1, -2, -3 and -8, Class II is represented by HDAC-4, -5, -6, -7, -9 and -10, Class III includes SIRT1–7, and Class IV comprises HDAC-11 [21,22]. According to the Human Protein Atlas, different HDAC family members are abundantly expressed in a variety of human cancers, such as hematological malignancies, breast, renal, thyroid, GI tract, skin, lung cancer and more. In head and neck tumors HDAC—Class I and II members’ expression (medium or high) ranges between ~25% to almost 100%, except HDAC-5, which is not detected in this group of cancers [23].

This study aimed to evaluate—for the first time to our knowledge—the HDAC-1, -2, -4 and -6 immunohistochemical expression in the group of rare and frequently challenging to manage SGTs. Furthermore, we aimed to investigate the possible correlations of HDAC-1, -2, -4 and -6 expression in malignant SGTs with clinicopathological characteristics and patients’ survival and their potential use as prognostic biomarkers.

## 2. Materials and Methods

### 2.1. Clinical Material

Medical records and archival histopathological material of 58 SGT patients were included in this study. All patients were diagnosed and underwent surgical therapy within the period 2002–2017. None of the patients received any treatment before surgery. According to standard histopathological protocols, the corresponding surgical specimens were examined at the Department of Pathology of the Institut Curie. All cases were reviewed and classified (by J.K. and S.T.) according to the latest World Health Organization Classification principles [24].

The study sample consisted of 36 benign and 22 malignant cases. The group of benign tumors incorporated 28 Pleomorphic adenomas (PA), 7 Warthin tumors (WT) and 1 Basal cell adenoma (BAD). The group of malignant tumors incorporated 3 Mucoepidermoid carcinomas (MEC, 1 LG and 2 HG cases), 4 Adenoid cystic carcinomas (ACC, all HG cases), 5 Acinic cell carcinomas (AcCC, 2 LG and 3 HG cases), 1 Basal cell adenocarcinoma (BAC, LG case), 1 Salivary duct carcinoma (SDC, HG by definition), 1 Epithelial-myoepithelial carcinoma (EMC, HG) and 7 Squamous cell carcinomas (SCC, all HG cases).

Eighteen (18) of the patients were male (31.03%), and 40 were female (68.97%), with a male to female ratio of 0.44 for the benign and 0.47 for the malignant SGT cases. Overall mean age at diagnosis was 57.72 years (ranging from 28 to 85 years) and 71.14 years (ranging from 41 to 93 years) for benign and malignant SGT patients, respectively.

During the observation period of patients with malignant SGTs, ranging from 10 to 170 months (median: 59.86 months), 6 patients died from their disease and 2 patients were alive with disease, whereas the remaining 14 patients were alive and free of disease.

### 2.2. Immunohistochemistry

Immunostainings for HDAC-1, -2, -4 and -6 were performed separately, on formalin-fixed, paraffin-embedded (FFPE) SGT tissue sections, using rabbit polyclonal anti-HDAC-1 (H-51, sc-7872, Santa Cruz Biotechnology, Santa Cruz, CA, USA), rabbit polyclonal anti-HDAC-2 (H-54, sc-7899, Santa Cruz Biotechnology), mouse monoclonal anti-HDAC-4 (A-4, sc-46672, Santa Cruz Biotechnology) and mouse monoclonal anti-HDAC-6 (D-11, sc-28386, Santa Cruz Biotechnology). In brief, 4 µm thick tissue sections were dewaxed in xylene and brought to water through graded alcohols. Antigen retrieval was performed by microwaving slides in 10 mM citrate buffer (pH 6.0) for 15 min at high power. To remove the endogenous peroxidase activity, sections were then treated with freshly prepared 3% hydrogen peroxide in methanol in the dark for 10 min at room temperature. Non-specific antibody binding was blocked using 5% normal goat serum (NGS) for 1 h. Tissue sections were incubated at 4 °C overnight against HDAC-1, -2, -4 and -6 diluted 1:100 in blocking buffer. Sections were then incubated at room temperature with biotinylated linking reagent (ab64264, Abcam, Cambridge, UK) for 10 min, followed by incubation with peroxidase-conjugated streptavidin label (ab64264, Abcam) for 10 min. The resultant immune peroxidase activity was developed using a diaminobenzidine (DAB) substrate kit (ab64264, Abcam, Cambridge, UK) for 10 min according to the manufacturer’s instructions. Sections were counterstained with Harris’ hematoxylin. Appropriate negative and positive controls were performed [25,26].

### 2.3. Evaluation of Immunohistochemistry

Immunohistochemical evaluation was performed by two independent pathologists (S.T. and D.P.) with no knowledge of clinical data. Samples were considered “positive” for HDAC-1, -2, -4 and -6 when >5% of tumor cells within the sample were stained. HDAC-1, -2, -4 and -6 immunoreactivity was estimated according to the percentage of positive tumor cells out of the total number of tumor cells within each specimen (0: negative staining, 0–4%, 1: 5–24%, 2: 25–49% and 3: 50–100%) and staining intensity was scored as 0: negative; 1: mild; 2: intermediate and 3: intense staining. The immunohistochemical H-score of HDAC-1, -2, -4 and -6 was calculated for the predominant staining intensity, using the formula “intensity score + percentage of tumor cells.” The expression of HDAC-1, -2, -4 and -6 was classified as low if the total score (*H* score) was 0–2 and high if the overall score was ≥3. In this way, we ensure that each group has a sufficient and more homogeneous number of comparable cases with the other groups [25,26]. 

### 2.4. Statistical Analysis

The associations between HDAC-1, -2, -4 and -6 expression (nuclear and/or cytoplasmic) with clinicopathological variables were calculated with Chi-square test, while their correlations with clinical outcome, as far as malignant cases are concerned, were assessed by constructing survival curves, using the Kaplan-Meier method. It has been previously reported that the critical decision making in both diagnosis and treatment of SGT should be based on the tumors’ histological subtype, and not on the specific pathological subtype. In this term, additional associations between benign and Low Grade (LG) and High Grade (HG) malignant cases have been conducted [27,28]. The differences between the curves were compared using the log-rank test. Results with a *p*-value of less than 0.05 were considered statistically significant. SPSS for Windows software was used for all analyses (SPSS Inc., V.25.0, Chicago, IL, USA).

## 3. Results

**Figure 1:**
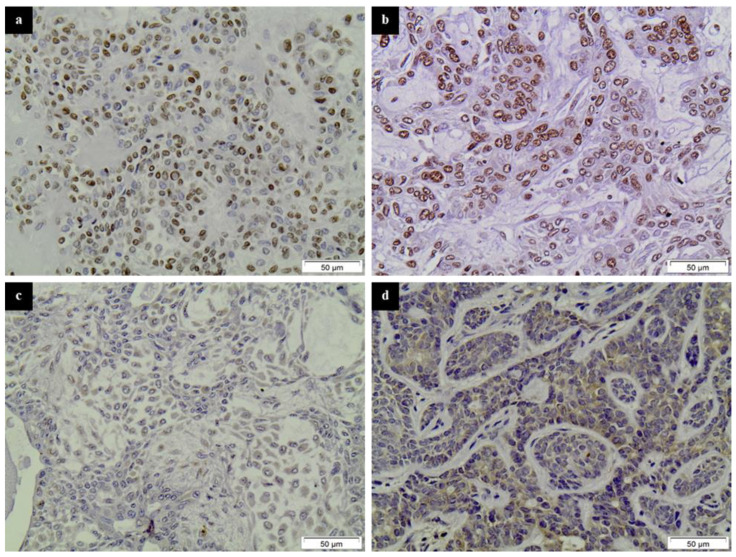
Representative immunostainings of HDAC-1, -2, -4 and -6 in benign SGTs. (**a**) HDAC-1 in a PA, (**b**) HDAC-2 in a PA, (**c**) HDAC-4 in a PA and (**d**) HDAC-6 in a BAD. Streptavidin-biotin-peroxidase, DAB chromogen, Harris hematoxylin counterstain.

**Figure 2:**
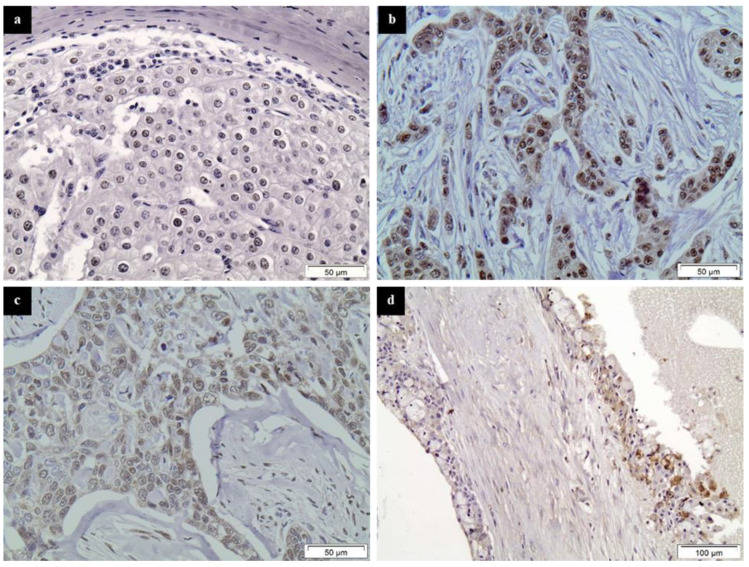
Representative immunostainings of HDAC-1, -2, -4 and -6 in malignant SGTs. (**a**) HDAC-1 in a HG ACC, (**b**) HDAC-2 in a HG SDC, (**c**) HDAC-4 in a HG SCC and (**d**) HDAC-6 in a HG MEC. Streptavidin-biotin-peroxidase, DAB chromogen, Harris hematoxylin counterstain.

All HDAC family members studied were abundantly expressed in SGTs (Figure 1 and Figure 2).

Class I HDAC-1 was expressed in about 31% of benign and 14% of malignant SGT cases with a nuclear distribution pattern. Class I HDAC-2 expression was detected in 86% of benign and 82% of malignant SGT cases. Interestingly enough, although the majority of tumors showed a nuclear staining pattern, cytoplasmic staining was also present in a few cases (2 PAs, 1 high-grade AcCC, 1 SDC and 1 SCC). Class IIa HDAC-4 stained 44% of benign and 36% of malignant tumors, mostly with a nuclear pattern, while 4 cases (different from the ones mentioned for HDAC-2) showed a cytoplasmic pattern of staining (3 PAs and 1 SCC). Finally, Class IIb HDAC-6 was expressed in 11% of benign and 18% of malignant SGTs, mainly with a cytoplasmic staining pattern. More details about the results of the immunohistochemical analysis of benign and malignant SGTs are presented in Table 1.

**Figure 3:**
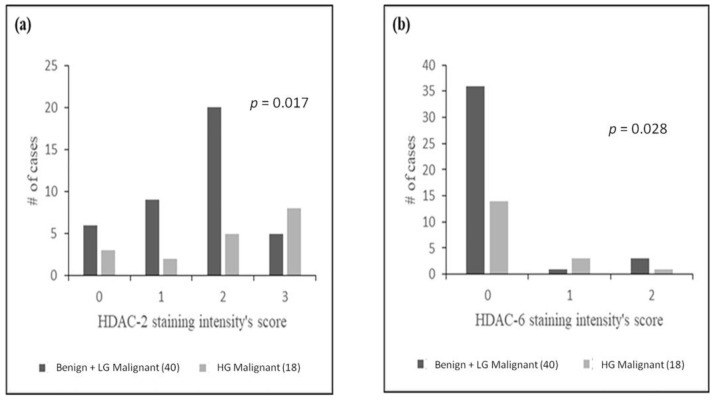
Correlation between staining intensity of (**a**) HDAC-2 and (**b**) HDAC-6 and benign plus LG malignant vs. HG malignant SGTs.

HDAC intensity of staining did not statistically differ between benign, LG malignant and HG malignant SGTs, probably since some of the 10 distinct SGT entities studied were not satisfactorily represented because of their rarity. However, when grouping benign and LG malignant SGT cases together, staining intensity for HDAC-2 and HDAC-6 successfully differentiated HG malignant from benign plus LG malignant tumors (*p* = 0.017 and *p* = 0.028, respectively) (Figure 3).

**Figure 4:**
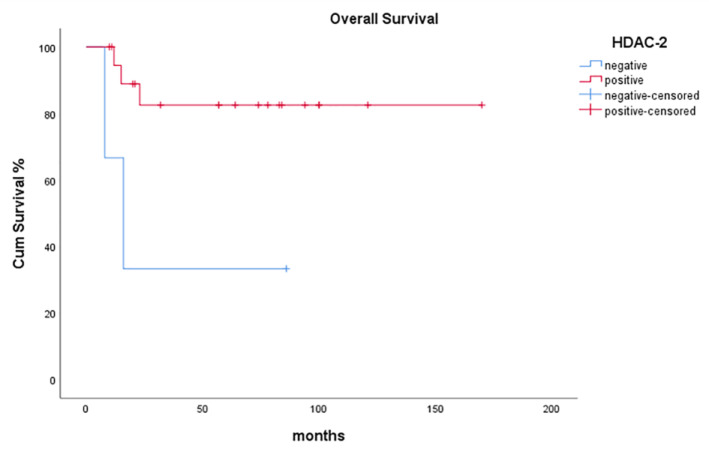
Kaplan-Meier survival analysis stratified according to HDAC-2 positivity in patients with malignant SGTs.

HDAC-2 positivity was significantly associated with more prolonged overall survival (OS) of patients with malignant SGTs (*p* = 0.028, Figure 4).

**Figure 5:**
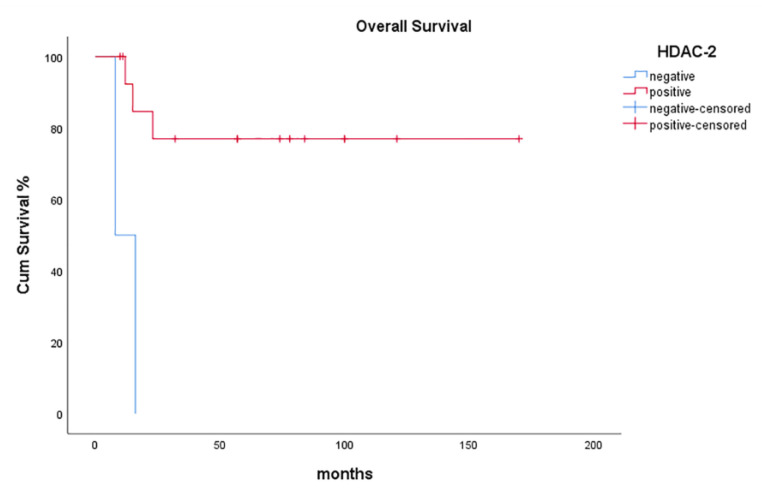
Kaplan-Meier survival analysis stratified according to HDAC-2 positivity in patients with HG malignant SGTs.

The favorable effect of HDAC-2 positivity was even more prominent for patients with HG malignant SGTs (*p* = 0.003, Figure 5).

**Figure 6:**
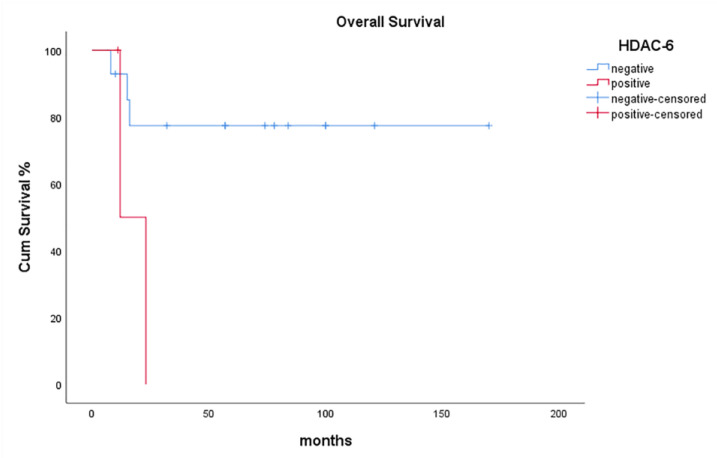
Kaplan-Meier survival analysis stratified according to HDAC-6 positivity in patients with HG malignant SGTs.

On the contrary, HDAC-6 positivity adversely affected OS of patients with HG malignant SGT, in the sense that no HDAC-6 expression was significantly associated with prolonged OS of these patients (*p* = 0.043, Figure 6).

## 4. Discussion

Several studies during the last two decades have provided evidence of HDAC overexpression in numerous human cancers, underlining significant correlations between high HDAC levels and clinicopathological parameters pivotal for accurate diagnosis and patients’ management, also providing prognostic information. In our study, which included 22 malignant SGT cases, HDAC-1, -2, -4 and -6 positivity was noted in 14% (3 out of 22), 82% (18 out of 22), 36% (8 out of 22) and 18% (4 out of 22), respectively. Additionally, high HDAC expression was noted in 33% (1 out of 3), 89% (16 out of 18), 62.5% (5 out of 8) and 25% (1 out of 4) of HDAC-1, -2, -4 and -6 positive SGTs, respectively (Table 1).

Although no statistically significant associations between HDAC expression and patients’ age and gender were recorded in our study, such correlations on different cancer types were found in other studies. More specifically, high HDAC-1 expression has been correlated with older patients’ age in gastric carcinoma [29] and with younger patients’ age and male gender in mobile tongue SCC [26]. In comparison, high HDAC-6 expression has been associated with younger patients’ age in invasive ductal breast carcinoma cases [30].

Positive correlations between high HDAC expression and tumor differentiation levels have also been assessed in numerous studies, especially Class I HDACs members. In our study, both Class I members, HDAC-1 and -2 and Class II members HDAC-4 and -6, were abundantly expressed in SGTs. The high number of distinct entities—most of them rare—studied herein (3 benign and 7 malignant histologic types of tumors) resulted in a disproportionate representation of each of them, posing restrictions on data analysis. However, HDAC-2 and HDAC-6 positivity were more frequently noted in HG SGTs when compared to the group of LG SGTs plus benign SGTs (*p* = 0.017 and *p* = 0.028, respectively). Our findings imply an inversely proportional association of HDAC-2 and -6 positivity with tumor differentiation grade.

HDAC-1 overexpression has been significantly associated with glioblastoma [31] (HG by definition) and poorly differentiated mobile tongue SCC [26], lip SCC [32], non-small cell lung (NSCLC) [33], hepatocellular [34], prostate [35,36], serous subtype of endometrial and ovarian [37] and urothelial bladder carcinoma [38]. On the contrary, Suzuki et al. [39], who have reported a gradual reduction of HDAC-1, -2, and -6 expression in the progression from healthy breast tissue to in situ and to invasive carcinoma, came up with a more significant HDAC-1 reduction in HG versus LG cases.

HDAC-2 overexpression has been correlated with HG lip SCC [32], NSCLC [33], esophageal [40], gastric [29,41], hepatocellular [34], prostate [35,36], serous subtype of endometrial and ovarian and urothelial bladder carcinoma [38], the latest more often accompanied by adjacent carcinoma in situ when HDAC-2 was overexpressed [37].

HDAC-3 overexpression has been shown in glioblastoma (grade IV by definition) [31] and poorly differentiated hepatocellular [34], prostate [35,36], serous subtype of endometrial and ovarian [37] and urothelial bladder carcinoma [38].

Observations regarding associations between HDAC family members’ overexpression and patients’ OS and/or DFS rates are also highly significant. In our study, after a mean follow-up period of 59.86 months, 14 patients with malignant SGT were alive and disease-free and 2 had a relapse, while 6 died of their disease. HDAC-2 positivity was significantly associated with prolonged OS of patients with malignant SGTs (*p* = 0.028). The association of HDAC-2 positivity and prolonged OS was even more prominent in HG malignant SGT cases (*p* = 0.003). On the contrary, HDAC-6 overexpression adversely affected HG malignant SGT patients since the absence of HDAC-6 expression was significantly associated with prolonged OS (*p* = 0.043).

HDAC-1 overexpression has been correlated with poor survival in mobile tongue SCC [25], gastric [29,41] and colorectal carcinoma [42], intrahepatic cholangiocarcinoma [43] and urothelial bladder endometrial and ovarian carcinoma [38], especially of endometrioid type [37,44]. Contrariwise, high levels of HDAC-1 expression have been connected with favorable prognosis in invasive breast [30,45] and pancreatic carcinoma [46].

HDAC-2 overexpression has been associated with poor prognosis in patients with gastric [41], colorectal [42], hepatocellular [34,47] and prostate carcinoma (especially Gleason score 7 cases) [35], while it has been connected with prolonged survival in invasive breast carcinoma cases [29].

HDAC-3 overexpression has been found to correlate with shorter survival rates in gastric [40], hepatocellular carcinoma [33,46] and glioblastoma [31], whereas it has been associated with prolonged survival in primary and stage IV metastatic melanoma cases [48].

No relevant data have been reported for HDAC-4, while high HDAC-6 expression has been emerging as a favorable prognostic factor in terms of survival in invasive breast [30] and pancreatic cancer [46].

Additionally, Class IIa members, HDAC-5 and -9, have been found to correlate with poor survival rates in medulloblastoma cases [49], while HDAC-7 overexpression has been related to significantly higher number of deaths and recurrences in pancreatic cancer [50].

In our study, tumor size for SGTs varied between 0.5 and 6 cm, lymph node metastasis was reported only in two cases, while there was no case with distant metastasis. Therefore, no significant correlations between tumor stage and HDAC subtypes overexpression could be identified. However, elevated HDAC expression has mainly been associated with advanced cancer stage in terms of tumor size, lymphatic/vascular invasion and presence of lymph node and/or distant metastases. 

HDAC-1 overexpression has been connected with advanced disease stage in cases of mobile tongue SCC [25], NSCLC [33], primary or recurrent gastric cancer [41,51,52] and intrahepatic cholangiocarcinoma [43]. On the contrary, heterogeneous HDAC-1 expression (high and low) has been correlated with lymph node metastasis in colorectal cancer [53].

HDAC-2 overexpression has also been associated with advanced disease stage in thyroid [26], NSCLC [33] and gastric cancer [41,54]. Additionally, high HDAC-2 expression, in terms of intense staining intensity, has been significantly correlated with muscular invasion and advanced depth of invasion in mobile tongue SCC cases [25].

HDAC-3 overexpression has been inversely associated with pT status in a cohort of renal carcinomas (RCC), especially of the papillary histologic type [55].

High HDAC-4 expression has been connected with capsular invasion in malignant thyroid lesions [26] and advanced-stage epithelial ovarian cancer [56]. On the contrary, high HDAC-4 expression has been correlated with the absence of organ and lymph node metastases in pancreatic adenocarcinoma [46].

HDAC-5 (as well as HDAC-1, -2, -3 and -4) expression in prostate carcinomas is two-fold higher in pT3 compared to pT2 cases and to connect with tumor recurrence and metastasis [57]. 

Finally, HDAC-6 overexpression has been connected with advanced stages (III and IV) in oral SCC cases [58]. Contrariwise, it has been associated with earlier histopathological stage and smaller tumor size in pancreatic adenocarcinoma cases [46]. 

Extensive research of HDACs’ impact on carcinogenesis has led to a group of potent epigenetic drugs. HDACs have already been approved for the treatment of cutaneous T-cell lymphoma, peripheral T-cell lymphoma and multiple myeloma. They are undergoing clinical trials—alone or in combination with other drugs—to treat numerous other cancers, such as lung, colorectal, cervical and ovarian, prostate and head and neck SCC [59]. Nevertheless, the studies using such drugs in SGT as a target for therapy are still limited [11].

Our study is the first to assess immunohistochemically the expression of HDAC family members in SGT tissue samples. Available data concerning the role of HDACs in SGTs remain scarce and HDACIs have been tried in SGTs without preceding evaluation of their expression levels. Wagner et al. [60] have recently immunohistochemically analyzed acetyl-histone H3 expression and Ki67 index in tissue microarrays (TMAs) of 84 cases of SGTs, providing evidence that malignant SGTs were hypo-acetylated compared to benign ones. They also showed that levels of acetyl-histone H3 were inversely correlated with Ki67 index of SGTs, suggesting that HDACs promote cell proliferation. Simultaneously, Ahn et al. [61] investigated the specific inhibition of HDAC-7 expression in apicidin (HDACI)-treated MEC cell lines, providing evidence that HDAC-7 downregulation inhibits cell proliferation and induces autophagy in MEC cells. Wagner et al. [62] also explored the benefits of HDACs and NFκB combined inhibition with the administration of Vorinostat (HDACI) and Emetine, respectively, in MEC cell lines, showing that Vorinostat efficiently disrupted the population of cancer stem cells (CSCs). Moreover, it failed in significantly reducing the total number of tumor cells. When combined, however, Vorinostat plus Emetine provided an effective regimen for managing MECs. Another contemporary study by Almeida et al. [63] investigated the effect of Vorinostat and cisplatin, alone or in combination on ACC cell lines, considering CSCs as a biological marker of therapy resistance in patient-derived xenograft (PDX) samples and ACC primary cells. Researchers concluded that Vorinostat’s administration reduces tumor cell viability and diminishes several detectable CSCs, with the effects being multiplied when combined with cisplatin. The same group of researchers had previously implied that sequential administration of Vorinostat and cisplatin (tow-hit regimen) minimizes the number of CSCs and prevents resistance in MEC cell lines [64]. The effect of chidamide, another HDACI, on human ACC cancer cells has also been assessed recently by western blot analysis of ACC cell lines [65]. Researchers presented evidence that chidamide induced histone-H3 acetylation resulting in diminished cell proliferation and cell-cycle arrest.

## 5. Conclusions

In our study, HDAC-2 expression emerges as an essential positive prognostic factor for SGT (good prognosis), whereas HDAC-6 expression as a negative one (poor prognosis). In addition, once the HDAC-6 overexpression is correlated with poor prognosis, an inhibitor of HDAC-6 may be used to improve patients’ survival. Although our work supports statistically significant results of clinical importance, the main criticism of such studies remains the small number of patients with rare neoplasms enrolled and the heterogeneity of the histological groups within studies. Further work, in larger cohorts of specific SGT histological subtypes, is mandatory to verify the essential prognostic role of HDACs in SGTs and/or unlock their potential as therapeutic targets, using HDACIs for SGT patients’ management. 

## Figures and Tables

**Table 1:** Detailed results of the immunohistochemical analysis of HDAC-1, -2, -4 and -6 expression in SGTs.

HDAC # OF CASES	HDAC-1		HDAC-2		HDAC-4		HDAC-6	
	Positive (%)	High *H*-Score (%)	Positive (%)	High *H*-Score (%)	Positive (%)	High *H*-Score (%)	Positive (%)	High *H*-Score (%)
TOTAL (58)	14 (24.14%)	4 (6.90%)	49 (84.48%)	44 (75.86%)	24 (41.38%)	13 (22.41%)	8 (13.79%)	4 (6.90%)
BENIGN (36)	11 (30.56%)	3 (8.33%)	31 (86.11%)	28 (77.78%)	16 (44.44%)	8 (22.22%)	4 (11.11%)	3 (8.33%)
PA (28)	10 (35.71%)	3 (10.71%)	26 (92.86%)	24 (85.71%)	15 (53.57%)	7 (25%)	3 (10.71%)	2 (7.14%)
WT (7)	1 (14.29%)	0 (0%)	4 (57.14%)	3 (42.86%)	0 (0%)	0 (0%)	0 (0%)	0 (0%)
BAD (1)	0 (0%)	0 (0%)	1 (100%)	1 (100%)	1 (100%)	1 (100%)	1 (100%)	1 (100%)
MALIGNANT (22)	3 (13.64%)	1 (4.55%)	18 (81.82%)	16 (72.73%)	8 (36.36%)	5 (22.73%)	4 (18.18%)	1 (4.55%)
MEC (3)	1 (33.33%)	1 (33.33%)	3 (100%)	3 (100%)	2 (66.67%)	2 (66.67%)	1 (33.33%)	0 (0%)
Low grade (1)	0 (0%)	0 (0%)	1 (100%)	1 (100%)	0 (0%)	0 (0%)	0 (0%)	0 (0%)
High grade (2)	1 (50%)	1 (50%)	2 (100%)	2 (100%)	2 (100%)	2 (100%)	1 (50%)	0 (0%)
ACC (4)	1 (25%)	0 (0%)	4 (100%)	4 (100%)	0 (0%)	0 (0%)	0 (0%)	0 (0%)
Low grade (0)	-	0 (0%)	-	-	-	-	-	-
High grade (4)	1 (25%)	0 (0%)	4 (100%)	4 (100%)	0 (0%)	0 (0%)	0 (0%)	0 (0%)
AcCC (5)	0 (0%)	0 (0%)	3 (60%)	2 (40%)	0 (0%)	0 (0%)	0 (0%)	0 (0%)
Low grade (2)	0 (0%)	0 (0%)	1 (50%)	0 (0%)	0 (0%)	0 (0%)	0 (0%)	0 (0%)
High grade (3)	0 (0%)	0 (0%)	2 (66.67%)	2 (66.67%)	0 (0%)	0 (0%)	0 (0%)	0 (0%)
BAC (1)	0 (0%)	0 (0%)	1 (100%)	1 (100%)	1 (100%)	1 (100%)	0 (0%)	0 (0%)
Low grade (1)	0 (0%)	0 (0%)	1 (100%)	1 (100%)	1 (100%)	1 (100%)	0 (0%)	0 (0%)
High grade (0)	-	-	-	-	-	-	-	-
SDC (1)	1 (100%)	0 (0%)	1 (100%)	1 (100%)	0 (0%)	0 (0%)	1 (100%)	0 (0%)
Low grade (0)	-	-	-	-	-	-	-	-
High grade (1)	1 (100%)	0 (0%)	1 (100%)	1 (100%)	0 (0%)	0 (0%)	1 (100%)	0 (0%)
EMC (1)	0 (0%)	0 (0%)	1 (100%)	1 (100%)	0 (0%)	0 (0%)	0 (0%)	0 (0%)
Low grade (0)	-	-	-	-	-	-	-	-
High grade (1)	0 (0%)	0 (0%)	1 (100%)	1 (100%)	0 (0%)	0 (0%)	0 (0%)	0 (0%)
SCC (7)	0 (0%)	0 (0%)	5 (71.43%)	4 (57.14%)	5 (71.43%)	2 (28.57%)	2 (28.57%)	1 (14.29%)
Low grade (0)	-	-	-	-	-	-	-	-
High grade (7)	0 (0%)	0 (0%)	5 (71.43%)	4 (57.14%)	5 (71.43%)	2 (28.57%)	2 (28.57%)	1 (14.29%)

## Data Availability

The data presented in this study are available on request from the corresponding author. The data are not publicly available due to ethical reasons.
